# Low-cost simulator for intra-abdominal bleeding

**DOI:** 10.1590/0100-6991e-20233512-en

**Published:** 2023-10-30

**Authors:** Camila Oliveira Fernandes, Lucas Ribeiro Rodrigues, Mattheus Lucca Batista Silva do Amaral, Sarah Jessica de Morais Rodrigues, Marcos Antonio Marton-Filho

**Affiliations:** 1 - Centro Universitário Estácio de Ribeirão Preto, Medicina - Ribeirão Preto - SP - Brasil; 2 - Universidade de São Paulo, Medicina - Bauru - SP - Brasil

**Keywords:** Education, Medical, High Fidelity Simulation Training, Hemostasis, Surgical, Low-cost Technology, Educação Médica, Treinamento com Simulação de Alta Fidelidade, Hemostasia Cirúrgica, Tecnologia de Baixo Custo

## Abstract

**Background:**

training in critical surgical situations is crucial for a safe outcome. The use of simulators is well established, although many are quite expensive, requiring the search for financially viable solutions for training centers.

**Methods:**

we built a low-cost simulator for intra-abdominal bleeding with inexpensive materials, such as a manikin chest, latex tubes, silicone rubber, and waterproof fabric, seeking to mimic the abdominal viscera and vessels and their anatomical correlations. An IV infusion set allowed simulated blood to flow under pressure, and the blood flowed freely during simulation. After obtaining a functional model, we selected general surgeons to validate the simulator and its use in teaching surgery. We used the content validity index (CVI), with a cutoff of 0.9.

**Results:**

the cost of building the prototype was US$71,00 in 2021, accounting for the purchase of the various necessary materials. Twelve raters participated in the validation tests. The results obtained from the feedback survey showed a good evaluation of all items, especially the recognition of the injured vessel, access to the vascular injury, hemostasis by manual compression, and hemostatic suturing.

**Conclusion:**

the proposed simulator obtained good results in scenarios of intra-abdominal bleeding from large vessels, as well as for hemostasis by manual compression and suturing. It proved to be a useful tool for training in critical intra- abdominal bleeding situations, while maintaining a low cost of building.

## RESUMO

**Introdução:**

o treinamento em situações críticas em Cirurgia é determinante para o desfecho seguro. O uso de simuladores é bastante consolidado, embora muitos apresentem custos muito elevados, sendo necessária a busca de soluções financeiramente viáveis para os centros de treinamento.

**Métodos:**

construímos um simulador de sangramento intra-abdominal de baixo custo com materiais simples como tronco de manequim, tubos de látex, borracha de silicone e tecido impermeável, buscando representar vísceras abdominais e vasos e suas correlações anatômicas. Um sistema de tubos e equipos de soro permitiu o fluxo, sob pressão, de sangue simulado, que vertia livremente durante a simulação. Após a obtenção de modelo funcional, selecionamos cirurgiões gerais para a validação do simulador e seu uso no ensino de Cirurgia. Utilizamos o Índice de Validação de Concordância (IVC), com corte de 0,9.

**Resultados:**

a construção do protótipo resultou em gasto de US$71,00 em valores de 2021, destinados à aquisição dos diversos materiais necessários. Doze avaliadores participaram dos testes de validação. Os resultados obtidos dos questionários demonstraram uma boa avaliação em todos os itens, destacando-se o reconhecimento do vaso traumatizado, o acesso à lesão vascular, o controle hemostático por pressão direta assim como por sutura direta da lesão.

**Conclusão:**

o simulador proposto obteve bons resultados em cenários de sangramento abdominal de grandes vasos, assim como o controle hemostático do mesmo por pressão direta e sutura. Mostrou-se ferramenta útil para a adaptação a situações de estresse no treinamento em sangramentos intra-abdominais, além de manter baixo custo de construção.

## INTRODUCTION

Bleeding after trauma is an important cause of morbidity and mortality^1^. Hemorrhage control is a challenge for surgeons, being an important cause of errors and preventable deaths^2^. Faced with the great difficulty of teaching technical skills and emotional control to young surgeons confronting major abdominal bleeding, the use of simulators is a valid and consolidated method in medical education. Among the simulators, there are different options as to technological complexity, fidelity, physical or virtual structure, and costs associated with their construction^3^ ^,^ ^4^.

The establishment of continuous simulation practices allied to the theoretical curriculum improves learning, both subjectively, that is, with students reporting improvement in their operational skills, and objectively^3^. There are different simulation models, some of high technology and complexity involving virtual tools, and some of human performance, both being expensive and available only in large centers. On the other hand, low-cost simulators (LCS) are a useful option for undergraduates and smaller training centers, as they reproduce surgical conditions with greater accessibility^5^. 

Practice in the simulator provides the transmission and acquisition of cognitive aspects, composed of fundamental basic knowledge and psychomotor facets, such as gestures, techniques, and procedures. This allows exposure to complex scenarios and procedures and self-assessment of one’s practice and proficiency. The training of skills in interpersonal relationships in terms of communication can also be included among the objectives of the simulation^6^ ^-^ ^8^.

We describe the development and content validation of an intra-abdominal bleeding simulator for hemostatic control training, which is low-cost, feasible, and reproducible, for teaching and developing surgical skills.

## METHODS

The model was built on an adult mannequin torso (72cm x 54cm) made of plastic material. The anterior part was replaced by a vinyl acetate (EVA) material plate added to a thin layer of sponge (72cm x 54cm), mimicking the skin and subcutaneous tissue, to allow the incision and access to the cavity.

### Construction of the simulator

The fixation of the structures on the mannequin started with the retroperitoneal structures. Initially, the entire back part of the mannequin was covered with a single layer of EVA to prevent liquid leakage during simulations. The vessels were made with latex tubes (measurements: Ref. 203 - 9mm external diameter, 6mm internal diameter) and distributed to represent aorta, vena cava, celiac trunk, superior and inferior mesenteric arteries, renal arteries and veins, and iliac arteries and veins.

**Figure 1:**
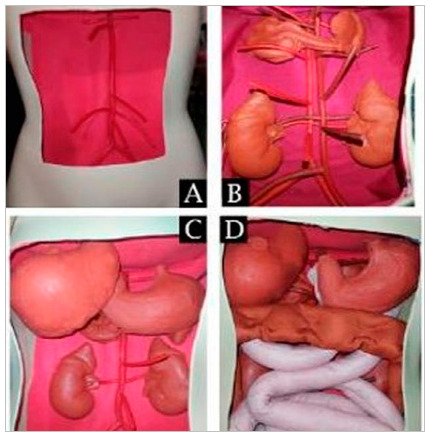
Attachment of structures to the simulator: blood vessels (A); retroperitoneal organs (B); liver and stomach (C); and intestines (D).

The main system, in which the simulated blood circulated, was composed only by the vena cava and aorta, which were the bleeding points in the proposed simulations. However, it could be directed to any of these vessels. In the arterial system, one end of the latex tube was occluded and the other attached to a system with a 1-liter saline bag filled with water with artificial red dye or artificial blood, on which rhythmic compressions were performed. In the venous system, one of the extremities was also occluded and the other attached to a 1-liter serum bottle, with a flow determined by gravity. The other vessels only created realism, but they were not filled with liquid. The choice was made due to the technical difficulty in executing multiple connections and the risk of leaks in the system (Figure 1A).

Still in the retroperitoneum, kidneys and pancreas were positioned, both made of silicone. The molds were obtained from commercial anatomical models. The organs of these models were isolated, immersed in negative plaster blocks for mold formation and, after drying and removing the original models, filled with silicone. The silicone models were then fixed in their anatomical position (Figure 1B). The peritoneal membrane was represented by a fine mesh of semitransparent fabric, isolating the peritoneal cavity from the retroperitoneum.

**Figure 2:**
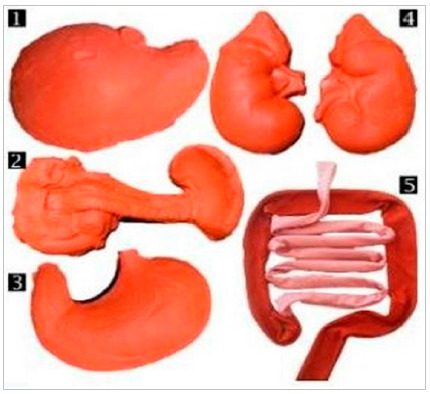
Made-up organs: liver (1), pancreas and spleen (2), stomach (3), kidneys (4), and small and large intestines (5).

Next, the peritoneal organs - liver, spleen, and stomach - were fixed using a silicone manufacturing process like that used for pancreas and kidneys (Figure 1C). The intestines were made of fine synthetic fabric mesh, with different colors for small and large ones. Both were filled with small amounts of Styrofoam microspheres to maintain their malleability, allowing easy handling. The intestines were fixed only at their ends, without the presence of the mesentery, as it could make it difficult to manipulate the structures and require greater maintenance of the simulator (Figure 1D). The organs are represented separately in Figure 2.

### Simulation Scenario

The evaluators were submitted to a simulated scenario using the simulator built in a previously prepared room of the Skills and Simulation Laboratory, with adequate focus of light, clothing, and instruments for laparotomy. The scenario model presented described a victim of an abdominal gunshot wound (GSW), with clear signs of hemodynamic instability, which unequivocally led to the indication of exploratory laparotomy.

**Figure 3:**
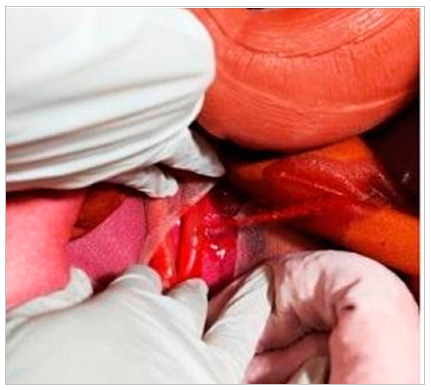
Focus of retroperitoneal bleeding.

The simulator was prepared with an injury to the abdominal aorta (partial section of the latex tube, measuring approximately 2cm) at the supramesenteric level and attached to serum bottles filled with simulated blood, allowing pulsatile flow in an adequate volume for vessel identification and suture (Figure 3).

**Figure 4:**
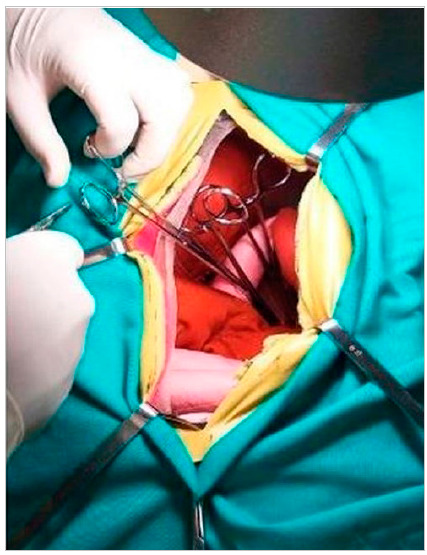
Simulated scenario using the simulator.

The evaluator should start the scenario by following all the laparotomy technical steps, from skin section, access to the cavity, identification of the bleeding point, digital control, and preparation for suturing the bleeding vessel. The patient presented progressive hemodynamic worsening (Figure 4).

### Validation

Validation of the simulator was carried out by volunteer surgeons, who responded to the invitation letter sent to all those who met the inclusion requirements, namely, experience in abdominal surgery or trauma surgery and an association with the university or its teaching hospital. Although the minimum number of evaluators for validation is controversial in the literature, some authors propose a minimum of five and a maximum of twenty participants for content validation^9^.

After the simulation, we applied a questionnaire with ten questions that dealt with reproducibility, surgical technique, educational usefulness, and experiences lived in the simulator by the research subject, with answers based on a modified Likert scale, graduated from one to four. Faced with the absence of a validated form that met the specific objectives of the study, the applied instrument was developed by the authors based on minimum expected skills and similar published works^5^ ^,^ ^13^.

We analyzed the data using the content validity index (CVI), which measures the proportion or percentage of evaluators who agree on pre-defined aspects of the questionnaire. We considered items valid when they presented a CVI >0.810.

The present study was registered and approved at Plataforma Brasil under opinion number 4,997,024.

## RESULTS

The construction stage of the simulator resulted in a final cost of US$ 71.00, maintaining the initial forecast and guaranteeing the viability of the low-cost proposal.

The maintenance required after the execution of each simulated scenario during the validation process generated an approximate cost of US$ 4.80. This value included small repairs needed in the simulator, replacement of single-use items, such as skin, and consumables, such as suture threads, saline solution, food coloring agent, and gauze.

The simulator has high durability. The materials that underwent repairs during the study were only the latex tubes, used to vent abdominal bleeding, and the textile mesh that simulated the peritoneum. Two replacements of these materials were necessary during the twelve carried out simulations.

Twelve of the invited surgeons (100%) accepted the letter of invitation and participated in all validation steps. Among them, three (25%) were specialists in trauma surgery and three (25%) in digestive system surgery, the others (50%) being general surgeons with experience in abdominal surgery. Regarding the time of experience in abdominal surgery, 75% of them had more than five years of experience.

The data from the questionnaires were compiled and summarized in Table 1, with the CVI presented as the sum of the agreement or disagreement items.

**Table 1:** Percentage of agreement of evaluators in the validation questions.

Modified Likert scale	Contributes to learning	Recommended for students and residents	Reproduces anatomical structures satisfactorily	Location of the injured structure	Simulates blood pressure	Possible access to the injured structure	Reproduces the consistency of human tissues	Hemostatic control by suture	Direct pressure control	Reproduces feelings of a hemorrhage scenario
I disagree	0%	0%	8.33%	8.33%	8.33%	8.33%	16.66%	16.66%	8.33%	0%
I agree	100%	100%	91.67%	91.67%	91.67%	91.67%	83.34%	83.34%	91.67%	100%

## DISCUSSION

The presented simulator is the result of several frustrated attempts and some successes, which we sought to detail in the method so that it is reproducible in several teaching and simulation centers. Low-cost templates allow broad access to high- quality training, and sharing solutions prevents repeating mistakes.

[Table t2]

**Table t2:** 

Materials	Value in reais (R$ unit)
1kg silicone rubber (2x)	119.80
Plaster (10kg)	30.00
Ballpoint pens (5 units)	10.00
Hydrographic Pen (6 units)	11.00
Hot glue (15 pcs.)	30.00
Sponge (8 blocks)	24.00
EVE	10.00
Waterproof fabric	15.00
Adult mannequin (torso)	50.83
Hot glue gun	30.00
Food coloring (5 units)	10.00
Latex Tube (4 units)	15.00
0.9% saline bag 1000mL	15.00
Saline infusion kit	2.50
TOTAL	BRL 373.13

In addition, the validation of the simulator by specialists, that is, its use in a teaching scenario of intra-abdominal hemorrhage control, provides greater confidence in how much the simulator can approach real situations and allows the end user to actually practice the expected skills. The CVI obtained on the addressed questions demonstrates the adequacy of this simulator in reproducing reality for training and allowing the development of skills and basic competences. Other validations are possible, such as its applicability in undergraduate classes, having the graduates themselves as judges.

We highlight the item “Reproduces feelings of a hemorrhage scenario”, with agreement of 100%, since attempts at creating reliable environments are constant in teaching practices^5^ ^,^ ^6^ ^,^ ^8^ ^,^ ^13^ ^,^ ^14^, which allow the reproduction of stress emotions and physiological reactions, especially in adverse and emergency scenarios. Thus, a great potential application of the simulator is the training of non-technical skills in crisis situations.

The items with the lowest agreement among the evaluators refer to the reliability of the material and its ability to reproduce human tissues. This is a common limitation in low-cost simulators, given that the materials are not created exclusively for this purpose, but various materials already available on the market are used. Literature data show, however, that this low similarity is not a limiting factor for its use in teaching^11^ ^,^ ^15^ ^-^ ^19^.

The items on contribution to learning and recommendation for students and residents lead us to reflect on the adequacy of the proposal for the curriculum of each institution. The simulator allows training of several skills, from the suture in depth, adding difficulty to technique training, to the construction of simulated scenarios for behavioral skills, which are more suitable for residents in the specialty.

Still regarding its applicability, considering the rapid expansion of laparoscopic and minimally invasive surgery, it is valid to assume that the model supports, with few adjustments, the adaptation of a laparoscopic training box, and may even expand its use to this access modality.

In addition to construction or acquisition, another factor that prevails in the real use of simulators is their maintenance and the number of simulations they allow until some repair is necessary. The average cost of validation scenarios was US$ 4.80, including consumables such as sutures. The value is still well below those practiced by major brands of simulators and is economically viable

## CONCLUSION

The making of the simulator involved the use of simple and low-cost materials, and its assembly is easily reproducible. The simulator can be used in numerous simulations, with little need for repairs, in addition to being easily transported and allocated in different environments.

In addition to manufacturing, we present data from validation of our model by experts. The good results obtained in the validation of this simulator by experienced surgeons reaffirm its didactic capacity.

Later validation steps with specific audiences will confirm its applicability in undergraduate and graduate education.
